# Empowering postpartum women through health education on Kegel exercises: effects on pain, pelvic floor dysfunction, and sexual function

**DOI:** 10.3389/frph.2026.1746383

**Published:** 2026-02-02

**Authors:** Jawaher H. Alharbi, Nesreen I. Abdul Manan, Neama Y. Hantira

**Affiliations:** 1 King Abdullah International Medical Research Center, Jeddah, Saudi Arabia; 2 King Saud bin Abdulaziz University for Health Sciences, College of Nursing, Jeddah, Saudi Arabia; 3 Ministry of the National Guard—Health Affairs, Jeddah, Saudi Arabia; 4 Faculty of Nursing, Alexandria University, Alexandria, Egypt

**Keywords:** Kegel exercise, pelvic floor dysfunction, postpartum complications, postpartum pain, sexual function

## Abstract

**Background:**

Strengthening the pelvic floor muscles through non-invasive Kegel exercises before the onset of clinical symptoms is the most effective method for reducing postpartum complications.

**Aim:**

This study seeks to investigate the impact of the Kegel exercise on maternal postpartum pain, pelvic floor dysfunction, and sexual function among women in the obstetrics and gynecology department of the National Guard Hospital, King Abdul-Aziz Medical City, Jeddah, Saudi Arabia.

**Methods:**

a quasi-experimental design with six weeks apart pre- and post-tests was used, having 31 participants per group recruited with a convenience sample method. An interviewer-administered questionnaire containing a 20-item Pelvic Floor Disability Index (PFDI-20), a 19-item Female Sexual Function Index (FSFI), and the Visual Analog Scale with the Faces Pain Rating Scale was used to measure pelvic floor dysfunction, sexual function, and postpartum pain, respectively. These tools were valid and reliable.

**Results:**

Results revealed a significant increase in the Female Sexual Function Index in the experimental group after the intervention and a substantial decrease in the Visual Analog Scale with the Face Pain Rating Scale in both groups after the intervention. There were no statistically significant differences between the experimental and control groups in the total score of pelvic floor dysfunction. However, the Colorectal-Anal Distress Inventory subscale showed a substantial increase in the control group post-test. In conclusion, the current study has shown that Kegel exercises can increase sexual drive and reduce pain in women with postpartum complications.

## Introduction

Childbirth can lead to complications such as pelvic floor weakness, pain, and ligament laxity, which may negatively affect women's sexual function and overall quality of life (1). Postpartum pain is a common complication and an important factor influencing various aspects of women's recovery, including physical function, psychological well-being, sleep, breastfeeding effectiveness, and bonding between mother and newborn (2). In a systematic review, Sultan and colleagues (3) reported that up to 18% of women experienced postpartum pain 12 months after a Cesarean section, while approximately 10% experienced persistent pain up to 18 months following any delivery method.

Another common postpartum complication is pelvic floor dysfunction (PFD), resulting from the impact of labor on pelvic muscle structures (1, 4). The pelvic floor muscles and connective tissues play a vital role in maintaining the stability of the pelvis, abdomen, and lower urinary tract (5). Consequently, these structures are susceptible to weakening or injury during labor and delivery, impairing their optimal function (6–8). In a meta-analysis, Barca and colleagues (9) reported that the most prevalent pelvic floor disorders among postpartum women were urinary incontinence (27.9%), pelvic organ prolapse (14.2%), and anal incontinence (0.4%). These complications could have serious impacts on the patient's body, mind, and social life and negatively impact their quality of life (1, 10–12).

Furthermore, postpartum sexual dysfunction is a notable complication often resulting from factors such as body image changes, fatigue, lack of sleep, and urinary stress or urge symptoms (13, 14). Sexual function typically declines after delivery and rarely returns to pre-pregnancy levels during the postpartum period, as many women report difficulties affecting sexual desire and relationships (13, 14). The prevalence of postpartum sexual dysfunction has been reported to range from 41% to 83% (13–15). In a study by Dawson and colleagues, nearly half of the participants experienced sexual dysfunction three months postpartum (16).

Medications and surgical interventions are available to manage postpartum discomfort, pelvic floor dysfunction, and erectile dysfunction (17). However, evidence indicates that pregnant and postpartum women should be encouraged to engage in exercise for both physical and mental well-being, with pelvic floor muscle training integrated into routine workouts to mitigate postpartum complications (18).

Several studies have shown that training the pelvic floor muscles can reduce the risk of postpartum issues and associated symptoms, provided that the exercises are initiated before clinical symptoms appear (19). This non-invasive intervention, known as Kegel exercise (20), involves repetitive contractions of the pelvic muscles that control urination, aiming to strengthen the pelvic floor, prevent or manage incontinence, reduce postpartum pain, and improve sexual responsiveness (21). Kegel exercises are also referred to as pelvic muscle strengthening exercises or pelvic floor exercises (PFE) (22, 23).

The researcher aims for this study to contribute to midwifery knowledge by highlighting how Kegel exercises can enhance the health and quality of life of postpartum women.

### Significance of the study

In Saudi Arabia, data on the prevalence of pelvic floor dysfunction remain limited, hindering the development of effective treatment strategies. Previous studies have assessed the benefits of Kegel exercises during pregnancy and for managing postpartum complications such as pelvic dysfunction and sexual impairment (18–20). However, a 2019 cross-sectional study in Madinah revealed that 57% of women had never practiced Kegel exercises, mainly due to inadequate educational programs (24). To date, no studies have evaluated the effects of these exercises on maternal postpartum pain, pelvic floor dysfunction, or sexual function among Saudi women. Consequently, the present study aims to examine these outcomes among women in Jeddah, providing essential evidence to support midwifery practice and enhance postpartum recovery and quality of life.

### Aim of the study

This study aims to identify the impact of the Kegel exercise on maternal postpartum pain, pelvic floor dysfunction, and sexual function among women in Jeddah, Saudi Arabia.

### Hypothesis

#### Null hypothesis

Training on Kegel exercises has no significant effect on pain, pelvic floor dysfunction, or sexual function among postpartum women.

### Conceptual framework

**Figure 1:**
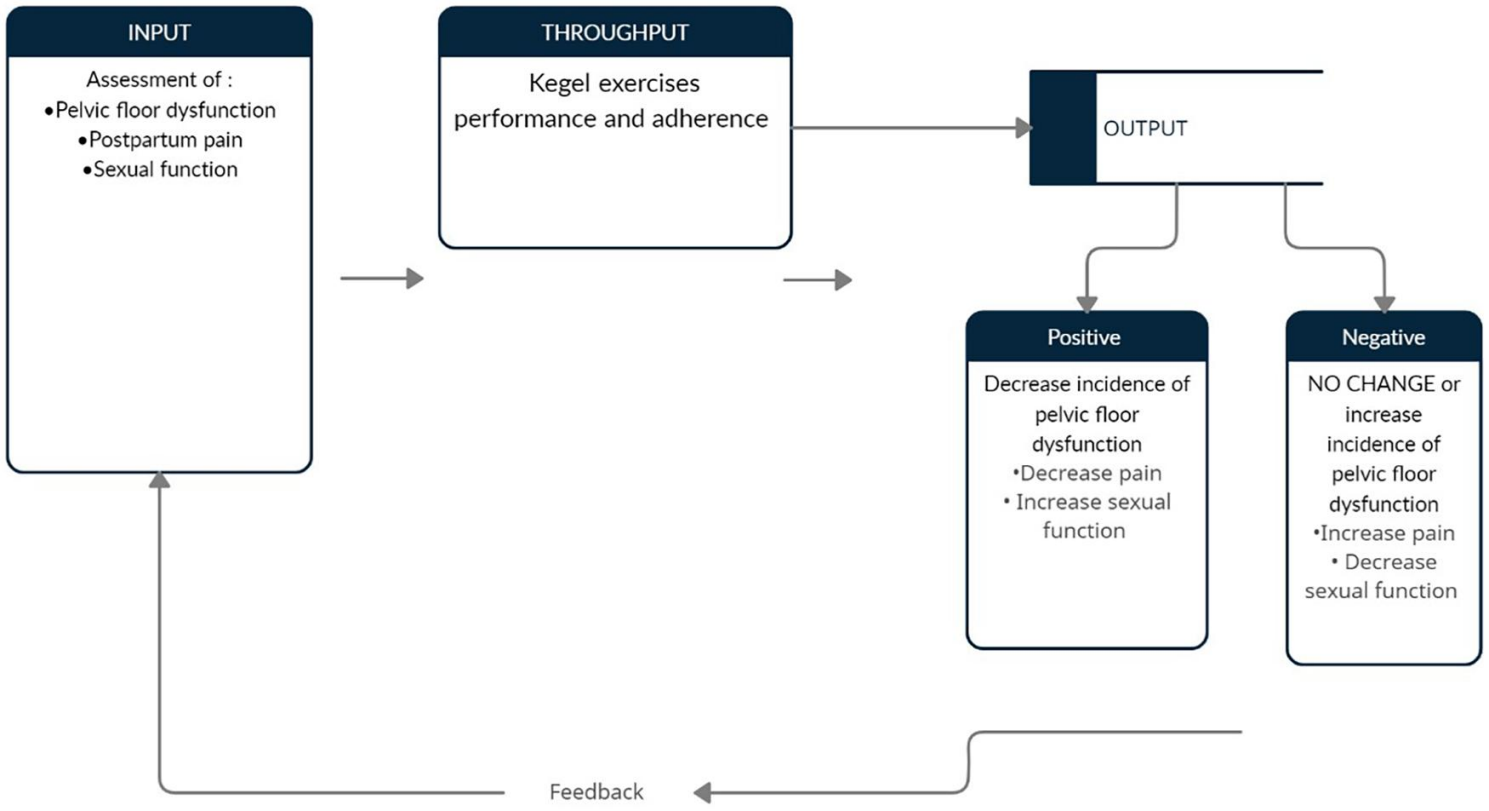
Conceptual framework based on general system theory of Ludwig Von Bertalanffy (25).

The General System Model, proposed by Ludwig von Bertalanffy (1968), provides the theoretical foundation for this study (Figure 1), emphasizing that systems consist of interrelated and dynamic components that interact with their environment (25, 26). Each system includes three key elements: input, throughput, and output, where inputs are transformed into outcomes through specific processes. Sustaining a system requires clear goals, feedback mechanisms, and adaptability to environmental changes (27). Within this study, the model is applied such that inputs represent the preparatory phase (interviews and assessments), throughput reflects the performance and adherence to Kegel exercises, and outputs denote the resulting maternal outcomes.

## Methodology

### Research design

This study used a quantitative quasi-experimental pre- and post-test design, which effectively tests cause-and-effect relationships and provides strong evidence of intervention efficacy (28). According to Shuttleworth, such designs are ideal for determining the extent of change resulting from an intervention (29). In this research, postnatal women were evaluated for pelvic floor dysfunction, sexual function, and pain before and after receiving training on Kegel exercises to assess their impact on these outcomes.

### Study area/setting

The postnatal women who participated in this study were recruited from the obstetrics and gynecology department of the National Guard Hospital located in King Abdul-Aziz Medical City in Jeddah, Saudi Arabia.

### Study population

The postnatal women in the obstetrics and gynaecology department in the King Abdul-Aziz Medical City, Jeddah, Saudi Arabia.

### Inclusion and exclusion criteria

Inclusion criteria include women who were ≤6 weeks postpartum, aged 18–45 years, multiparous, had a vaginal delivery, and were able to communicate in Arabic or English. Exclusion criteria included women with urinary or gastrointestinal infections, inflammatory conditions, significant comorbidities, or cognitive and behavioral impairments, to prevent potential confounding effects on the study outcomes.

### Sampling and sampling technique

The sampling technique adopted for this study is the convenience sampling method to recruit study subjects who have given their consent and met the study's inclusion criteria after institutional review board approval is obtained from the King Abdullah International Medical Research Center. The sample was randomly allocated into control and experimental groups using SPSS software as a simple randomization allocation.

### Sample size

Using G*Power 3.1.9.7, the necessary sample size was determined. An earlier Cochrane evaluation estimated the effect size of Kegel and other pelvic floor muscle training for preventing and treating mild problems in pregnant and postpartum women (30). With an alpha of 0.05 and a substantial effect size of 0.88, the initial power analysis predicted that 31 subjects per group would be needed to achieve 95% statistical significance (30). However, one experimental study subject withdrew during the study.

### Research tools

#### Pelvic floor dysfunction (PFD) assessment/pelvic floor disability index (PFDI-20)

This tool was used by Artymuk and Khapacheva (19). The Pelvic Floor Disability Index (PFDI-20) was adapted from the Pelvic Floor Disability Inventory (PFDI). It is a questionnaire designed to evaluate the health-related quality of life of women who experience pelvic floor disorders (31). The PFDI-20 consists of three sub-scales: The Urinary Distress Inventory-6 (UDI-6), the Colorectal-Anal Distress Inventory-8 (CADI-8), and the Pelvic Organ Prolapse Distress Inventory-6 (POPDI-6). The questions on the PFDI-20 are preceded by a response of either yes or no. If the patient answers “yes,” then they will be asked to rate the severity of their bowel, bladder, or pelvic symptoms that they have experienced over the course of the past three months on a scale that ranges from “not at all” (0) to “quite a little” (4). The individual scale scores are determined by multiplying the mean of the items that make up the scale by 25. The possible scale scores range from 0 to 100. The total score on the PFDI-20, which ranges from 0 to 300, is determined by summing the scores on the three individual subscales. A score of 300 signifies high distress, with a lower score suggesting less distress (31–33).

#### Female sexual function index (FSFI)

The FSFI is a 19-item self-report tool that evaluates female sexual function, as used in prior investigations (19, 34). It is made up of six different categories, which are as follows: desire (2 items), lubrication (4 items), orgasm (3 items), arousal (4 items), satisfaction (3 items), and pain (3 items). The items relating to desire and satisfaction are rated on a Likert scale with five points, ranging from one to five, while the remaining items are rated on a Likert scale with six points, ranging from zero to five. The range of total scores is from 2 to 30, with lower numbers indicating less functional sexuality (35). In this study, two domains were selected to be assessed among the sample, which include: Arousal and Desire domains, with 6 items; particularly in conservative cultural contexts, to minimize participant discomfort, only the desire and arousal domains of the FSFI were used, as these were deemed the most culturally acceptable.

#### Pain and functional disability assessment

**Figure 2:**
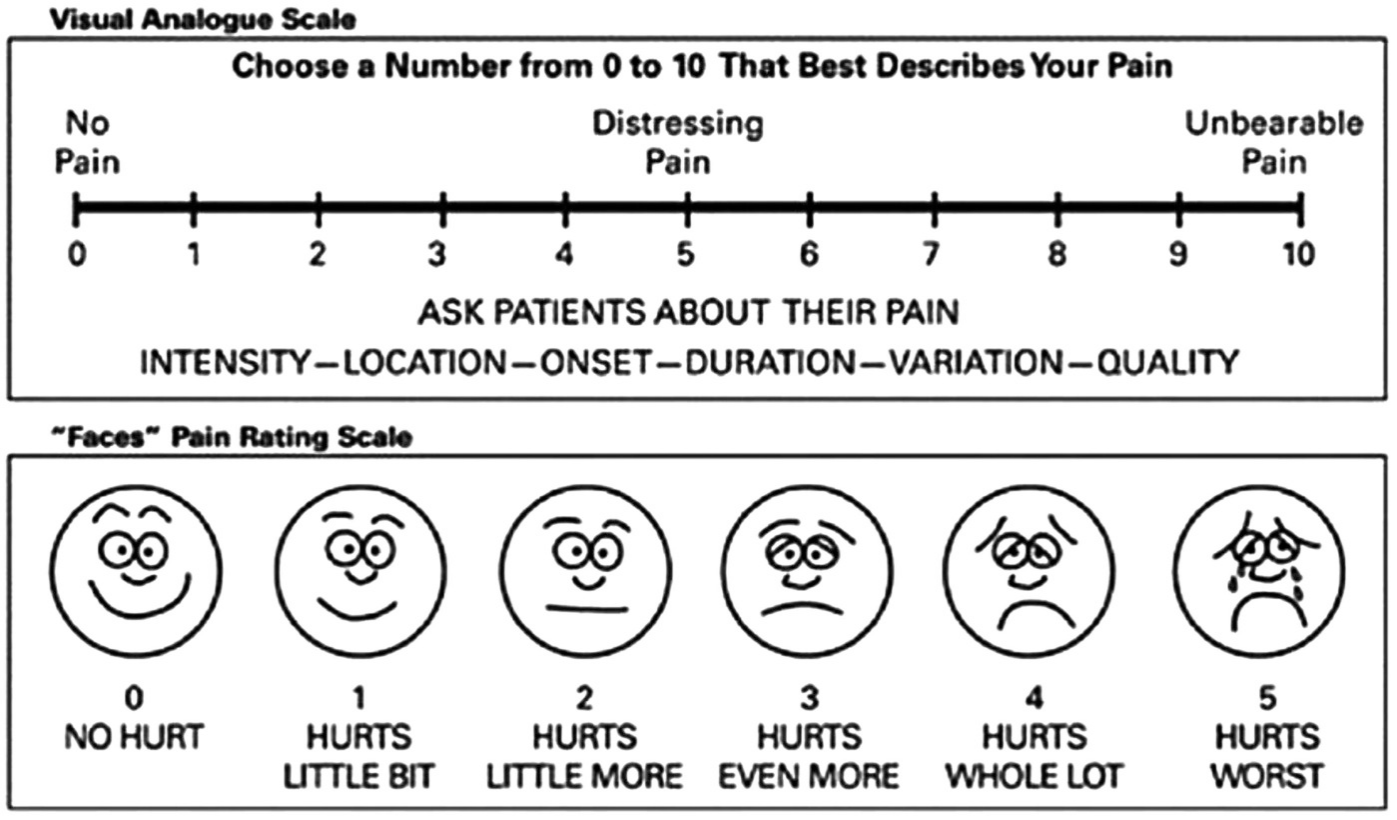
Visual analog scale (VAS) (6).

The Visual Analog Scale (VAS) instrument was developed by Hayes & Patterson in 1921, and it is commonly utilized to evaluate states such as anxiety, despair, weariness, and pain (6). A VAS is also the method most often used to determine the level of itching. The VAS is a visual aid that consists of a horizontal line that is 100 mm long. One end of the line is labeled “no pain,” while the other end is labeled “unbearable pain” (Figure 2). The patient is instructed to choose a number to describe the pain's intensity. The scoring criteria were from 0 for no pain to 10 for the worst imaginable pain.

### Data collection procedure

Data were collected using an interviewer-administered questionnaire describing the sociodemographic, obstetric characteristics, Pelvic Floor Disability Index, Female Sexual Function Index, and the Pain and Functional Disability Assessment of respondents.

The data collection was conducted through three phases:

First: preparation phase: Here, institutional review board approval was obtained from the King Abdullah International Medical Research Center, and the participating women's written consent was obtained. Orientation about the study to participants was provided, ensuring it was standardized for all participants; their contact number was collected, and social network groups were created.

Second: Intervention Implementation Procedures: Participants completed questionnaires for a pre-test. The experimental group received an orientation on Kegel exercise training, supported by a video demonstrating the function of the pelvic floor muscles and Kegel procedures, as adopted from Gustirini et al. (36). After that, participants were given a Kegel training DVD to watch at home and were instructed to perform the exercises daily, at least 3–4 times per day. The researcher maintained weekly follow-up via WhatsApp group for six weeks to answer any questions regarding the exercises and to conduct surveys (30). In contrast, the control group was contacted only once every two weeks for six weeks to provide counseling and encourage engagement in physical activity.

Third: Evaluation Phase: The Pelvic Floor Disability Index, Female Sexual Function Index, and pain assessment questionnaires were completed as a post-test for all participants (after six weeks) through an electronic link to the questionnaire via WhatsApp for the two groups.

### Data analysis

Using the International Business Machines-Statistical Package for Social Sciences (IBM-SPSS) version 29 for Windows, the obtained data were coded, entered, and analyzed. The demographic data's descriptive statistics were analyzed using frequency, percentages, and mean analyses. The Univariate Analysis of Variance (ANCOVA) and Student's *t*-test were used to determine any significant differences between the study groups on the study variables. Repeated measures multivariate analysis of variance (MANOVA) was used to analyze within and between-group differences of all variables in both groups. *P*-value < 0.05 was considered significant. Effect size (Cohen's d) was calculated as the between-group difference divided by the pooled standard deviation at baseline. Effect sizes of 0.20, 0.50, and 0.80 were considered small, moderate, and large, respectively (Suggested by Cohen). A Pearson's Correlation was run to determine the relationship between pre- and post-tests for the four domains and participants’ personal data and pregnancy history.

### Validity and reliability

To ensure the reliability and validity, relevance, language clarity, and applicability of the study tool were tested. The coefficient alpha (or Cronbach's alpha) is the most commonly used statistic for measuring internal consistency (37). In this study, the Cronbach's alpha coefficient was calculated by using SPSS version 29, using this formula for the Cronbach's alpha: $\alpha =\frac{N\bar{c}}{v+(N-1)\bar{c}}$ Here *N* is equal to the number of items, $\bar{c}$ is the average inter-item covariance among the items, and $\bar{v}$ equals the average variance. A Cronbach's alpha value of 0.88 and 0.93 was obtained for the PFDI-20 and FSFI tools, respectively. Generally, a score of more than 0.7 is considered acceptable.

The study also employed construct and face validity. The research instrument's face validity was ensured through a questionnaire review with two senior lecturers. After the completion of the questionnaire, construction of its items, and presenting it to the research supervisor's Excellency, the questionnaire was translated into the Arabic language and presented in its modified form to a group of arbitrators, and after retrieving the arbitrated version, their notes were discussed with the supervisor's Excellency of the thesis. Considering some arbitrators’ suggestions, the researcher reworded the questionnaire, where some of the coefficient values were high, indicating the availability of a high degree of internal consistency validity for the questionnaire's items.

### Ethical consideration

This research was authorized by the Institutional Review Board (IRB) at King Abdul-Aziz Medical City-Western Region and the College of Nursing Research Unit of the King Abdullah International Medical Research Center (KAIMRC) [IRB/1251/22, study number SP22J/060/05], Jeddah. Additionally, hospital administrative personnel were contacted before data collection, written informed consent was obtained from participants who met the criteria for inclusion to participate in the current study, and participation was voluntary. Furthermore, privacy, confidentiality, and anonymity were maintained.

### Trial registration

This study adopted a quasi-experimental design to assess the effect of Kegel exercises on postpartum outcomes. As the intervention was behavioral and non-pharmacological, it did not meet the criteria for a clinical trial requiring prospective registration according to WHO guidelines. The study protocol was approved by the Institutional Ethical Committee (Approval No.: [IRB/1251/22, study number SP22J/060/05), and written informed consent was obtained from all participants before data collection.

## Results

In this study, the mean age of the control group is 30.13 **±** 5.46, and the experimental group is 30.74 **±** 5.37. Almost all (control group = 100%, experimental group = 96.7%) of the studied women were married. Also, it was noted that more than two-thirds of the sample (control group = 67.7%, experimental group = 73.3%) held a bachelor's degree, and one quarter (control group = 25.8%, experimental group = 23.3%) of the women finished high school. Also, the table presents that less than three quarters (control group = 67.7%, experimental group = 73.3%) of the women were non-working, whilst more than one quarter (control group = 32.3%, experimental group = 26.7%) of them were working. More than half (control group = 58.1%, experimental group = 50%) of the women reported an average monthly income, and almost all the sample was from an urban place of residence (control group = 90.3%, experimental group = 93.3%). Also, the Unpaired *t*-test showed a non-significant difference (*p* > 0.05) between the two groups in age. Furthermore, the table demonstrated that there were no statistically significant differences between the two study groups regarding their sociodemographic data (Table 1).

**Table 1:** Distribution of the study groups (control and experimental group) according to their demographic and personal characteristics (*n*.61).

Demographic and personal characteristics		Groups				Test of Significance (*P*-value)
		Control group		Experimental group		
		*n*.31	%	*n*.30	%	
Age						t = −.446 (0.657)
Mean ± SD		30.13 ± 5.46		30.74 ± 5.37		
Min-max		20–40		21–40		
Marital status	Married	31	100.0%	29	96.7%	X^2^ = 1.016 (.313)
	Divorced	0	0.0%	1	3.3%	
Education	Secondary	1	3.2%	0	0.0%	X^2^ = 1.023 (.796)
	High school	8	25.8%	7	23.3%	
	Bachelor's degree	21	67.7%	22	73.3%	
	High degrees	1	3.2%	1	3.3%	
Working condition	Working	10	32.3%	8	26.7%	X^2^ = .313 (.576)
	Non-working	21	67.7%	22	73.3%	
Monthly income	Enough	11	35.5%	15	50.0%	X^2^ = 1.221 (.543)
	Average	18	58.1%	15	50.0%	
	Insufficient	2	6.5%	0	0.0%	
Place of residence	Rural	3	9.7%	2	6.7%	
	Urban	28	90.3%	28	93.3%	

Descriptive statistics, *t*, *t*-test; ×^2^, chi-square, *p* ≤ 0.05.

As shown in Table 2 below, more than half of the control group (58.1%) and less than three-quarters (70.0%) of the experimental group had no miscarriages. Whilst more than one-third (32.3%) of the control group and more than one-quarter (26.7%) of the experimental group had one to three miscarriages. Regarding the type of delivery of the women's last birth, more than three-quarters of the control and the experimental groups gave birth to their last child vaginally (77.4% and 83.3%, respectively). Less than one-quarter of the control and the experimental groups gave birth to their last child by an interventional delivery (22.6% and 16.7%, respectively). Also, the Unpaired *t*-test showed no significant difference (*p* > 0.05) in the number of children between the two groups. There were no statistically significant differences between the two study groups regarding their previous pregnancy history (Table 2).

**Table 2:** Distribution of the study groups (control and experimental group) according to their previous pregnancy history (*n*.61).

Previous pregnancy history		Groups				Significant differences tests
		Control group		Experimental group		
		*n*.31	%	*n*.30	%	Fisher-exact test (sig.)
Miscarriage	No miscarriage	18	58.1	21	70.0	FET = 1.387 (0.749)
	1–3 miscarriage	10	32.3	9	26.7	
	>3 miscarriage	3	9.7	1	3.3	
Type of delivery	Vaginal delivery	24	77.4	26	83.3	FET = 1.437 (0.503)
	Interventional/instrumental delivery	7	22.6	5	16.7	
Number of children						
Mean ± SD		3.35 ± 1.78		2.90 ± 1.56		*t*-test (*p*-value) −1.059 (.294)
Min-max		1.00–8.00		1.00–7.00		

Descriptive statistics, *t*, *t*-test, FET, fisher-exact test, *p* ≤ 0.05.

Table 3 presents the Item-level descriptive analysis of the PFDI-20, showing mild to moderate pelvic floor symptoms at baseline in both groups. After Kegel exercise training, the experimental group demonstrated numerical improvements in several prolapse-related, colorectal-anal, and urinary symptoms. However, similar variations were also observed in the control group, suggesting natural postpartum recovery. Given the small magnitude of change and overlapping variability, item-level findings were interpreted cautiously, and conclusions regarding intervention effectiveness were based primarily on domain-level and total PFDI-20 scores, which offer greater clinical and statistical robustness.

**Table 3:** Descriptive item-level distribution of pelvic floor disability Index (PFDI-20) symptoms At baseline and after Kegel exercise training Among the control and experimental groups.

Pelvic floor disability index (PFDI-20)	Time point	Control group (n.31) Mean ± SD	Experimental group (n.30) Mean ± SD
Pelvic organ prolapse distress inventory-6 (POPDI-6)			
1. Pressure in the lower abdomen	Baseline	.9 ± 1.4	1 ± 1.3
	After training	.7 ± 1.2	1.2 ± 1.2
2. Heaviness/dullness in the pelvic area	Baseline	1.2 ± 1.4	1.3 ± 1.3
	After training	1.0 ± 1.3	1.0 ± 1.1
3. Bulge/prolapse sensation	Baseline	.8 ± 1.2	.6 ± 1.1
	After training	.9 ± 1.5	.8 ± .9
4. Push the vagina/rectum for bowel movement	Baseline	1.3 ± 1.3	1.0 ± 1.3
	After training	.9 ± 1.3	1.1 ± 1.2
5. Incomplete bladder emptying	Baseline	1.3 ± 1.4	1.2 ± 1.3
	After training	.6 ± 1.0	1.0 ± 1.1
6. Push up bulge to urinate	Baseline	.5 ± 1.0	.5 ± .8
	After training	.3 ± .8	.5 ± .8
Colorectal-Anal Distress Inventory subscale (CRAD)			
7. Straining during bowel movement	Baseline	1.5 ± 1.4	1.1 ± 1.3
	After training	.8 ± 1.3	1.3 ± 1.3
8. Incomplete bowel emptying	Baseline	1.3 ± 1.3	1.0 ± 1.1
	After training	1.0 ± 1.3	1.3 ± 1.4
9. Stool leakage (solid)	Baseline	.8 ± 1.2	.6 ± 1.0
	After training	.5 ± 1.0	1.0 ± 1.2
10. Stool leakage (loose)	Baseline	1.0 ± 1.2	.6 ± 1.0
	After training	.5 ± 1.0	.9 ± 1.1
11. Gas leakage	Baseline	1.3 ± 1.2	.9 ± 1.0
	After training	.8 ± 1.3	1.3 ± 1.3
12. Pain with bowel movement	Baseline	1.1 ± 1.2	.7 ± 1.0
	After training	1.0 ± 1.4	1.4 ± 1.3
13. Fecal urgency	Baseline	1.1 ± 1.3	.8 ± 1.0
	After training	.8 ± 1.3	1.2 ± 1.2
14. Rectal prolapse sensation	Baseline	.8 ± 1.2	.5 ± .8
	After training	.4 ± .9	.9 ± 1.2
Urinary distress inventory-6 (UDI-6)			
15. Frequent urination	Baseline	1.1 ± 1.0	1.4 ± 1.3
	After training	.8 ± 1.3	1.4 ± .9
16. Urgency-related leakage	Baseline	1.1 ± 1.2	1.2 ± 1.2
	After training	.7 ± 1.2	1.1 ± .9
17. Stress urinary leakage	Baseline	1.0 ± 1.1	1.0 ± 1.1
	After training	.8 ± 1.1	1.2 ± 1.0
18. Small urine leakage	Baseline	.8 ± 1.1	1.0 ± 1.1
	After training	.6 ± 1.0	1.0 ± 1.0
19. Difficulty emptying bladder	Baseline	.8 ± 1.1	.8 ± 1.1
	After training	.5 ± 1.1	1.0 ± 1.0
20. Lower abdominal/genital pain	Baseline	.9 ± 1.1	.9 ± 1.3
	After training	1.0 ± 1.3	1.4 ± 1.1

Table 4 summarises the mean values of all PFDI-20 subscales at baseline and after intervention in both groups. The *t*-test revealed statistically significant differences in the Colorectal-Anal Distress Inventory (CRAD) between the control and experimental groups at *P* = 0.014. It was also identified that there was a significant increase in CRAD scores in the control group after the test, with a mean score of 28.73 ± 26.47. ANCOVA showed no statistical differences between the experimental and control groups in the total score of pelvic floor dysfunction (Table 3).

**Table 4:** Univariate analysis of variance (ANCOVA) and independent samples *t*-test for PFDI-20 subscales in both groups (control and experimental).

Domains		Maximum allowed score	Group		Significance test independent samples *t*-test		Univariate analysis of variance (ANCOVA)
			Control group *n*.31	Experimental group *n*.30			
			M ± SD	M ± SD	t	*p*-value	
Pelvic organ prolapse distress inventory (POPDI)	Baseline	100	22.58 ± 22.30	24.86 ± 24.92	−1.096	.277	F = 3.368 *p*-value = .072
	After Kegel exercise training		23.39 ± 20.46	18.19 ± 26.11			
Colorectal-anal distress inventory (CRAD)	Baseline	100	18.45 ± 19.55	28.12 ± 25.27	−2.531	.014*	
	After Kegel exercise training		28.73 ± 26.47	17.81 ± 25.59			
Urinary distress inventory 6 (UDI-6)	Baseline	100	26.34 ± 26.14	23.19 ± 22.58	−21.148	.256	
	After Kegel exercise training		29.70 ± 20.38	18.33 ± 25.79			
Pelvic floor disability index (PFDI)	Baseline	300	67.37 ± 60.14	76.18 ± 66.70	1.845	.070	
	After Kegel exercise training		81.82 ± 59.81	54.34 ± 74.23			

Descriptive statistics, *t*, *t*-test; F, ANCOVA; NB, The higher mean means an increase in pelvic floor disability.

* Significant at *p* ≤ 0.05.

Table 5 shows the Item-level FSFI analysis, which showed mild to moderate sexual desire and arousal at baseline with comparable scores between groups. After training, the experimental group exhibited modest and variable numerical changes, while the control group showed increases across several items, likely reflecting natural postpartum recovery. Given the small changes and overlapping variability, item-level findings were interpreted descriptively, and conclusions regarding intervention effects were based on domain-level and total FSFI scores.

**Table 5:** Descriptive item-level distribution of female sexual function Index (FSFI) desire and arousal items At baseline and after Kegel exercise training Among the control and experimental groups.

Female sexual function index (FSFI) (over the past 4 weeks)	Time point	Control group (*n*.31) Mean ± SD	Experimental group (*n*.30) Mean ± SD
Desire			
Frequency of sexual desire or interest	Baseline	2.5 ± 1.2	3.0 ± 1.0
	After training	3.2 ± 1.0	2.7 ± 1.1
Level (degree) of sexual desire or interest	Baseline	3.0 ± .8	2.8 ± .8
	After training	3.4 ± 1.0	2.5 ± .8
Arousal			
Frequency of sexual arousal (“turned on”)	Baseline	2.8 ± 1.3	2.8 ± 1.2
	After training	3.3 ± 1.2	2.3 ± .9
Level of sexual arousal (“turn on”)	Baseline	2.9 ± .8	2.7 ± .9
	After training	3.4 ± .8	2.8 ± .6
Confidence in becoming sexually aroused	Baseline	3.1 ± 1.1	3.2 ± .9
	After training	3.4 ± 1.3	2.8 ± .9
Satisfaction with sexual arousal	Baseline	3.3 ± 1.3	3.3 ± 1.2
	After training	3.4 ± 1.2	2.7 ± .9

Furthermore, Table 6 presents the mean values of all Female Sexual Function Index (FSFI) domains at baseline and after intervention in both groups. *T*-tests for both groups specified that there were statistically significant differences in desire and arousal domains of the Female Sexual Function Index (FSFI) (.009,.005, and.002), respectively. They illustrated that higher scores in both FSFI domains, Desire and Arousal, were indicated for the experimental group after the Kegel exercise training intervention, with a mean of (3.92 ± 1.11, 4.08 ± 1.22), respectively. Moreover, ANCOVA showed that the differences between the control and experimental groups are statistically significant in FSFI (F = 12.333, *p*-value = .001*) (Table 6).

**Table 6:** Univariate analysis of variance (ANCOVA) and independent samples *t*-test for FSFI domains in both groups (control and experimental).

The female sexual function index		Maximum allowed score	Group		Significance test independent samples *t*-test		Univariate analysis of variance (ANCOVA)
			Control group M ± SD	Experimental group M ± SD			
					T	p	
Desire	Baseline	6	3.50 ± .90	3.30 ± 1.16	2.691	.009*	F = 12.333 *p*-value = .001*
	After Kegel exercise training		3.12 ± 1.03	3.92 ± 1.11			
Arousal	Baseline	6	3.64 ± 1.06	3.61 ± 1.19	2.908	.005*	
	After Kegel exercise training		3.18 ± .87	4.08 ± 1.22			
The female sexual function index (FSFI)	Baseline	12	7.14 ± 1.72	6.91 ± 1.97	3.168	.002*	
	After Kegel exercise training		6.30 ± 1.74	8.00 ± 2.21			

Descriptive statistics, *t*, *t*-test; F, ANCOVA.

* Significant at *p* ≤ 0.05.

Table 7 presents the mean values of the pain scales at baseline and after intervention in both groups. *T*-test illustrated that there were statistically significant differences between control and experimental groups after intervention in the Visual Analog Scale (VAS) and Faces Pain Rating Scale at *p* < 0.05. Also, the *t*-test showed a significant decrease for the experimental group after the Kegel exercise intervention in both subscales (2.40 ± 2.16 &83 ± 1.02). ANCOVA showed that the differences between the control and experimental groups were statistically significant in the Visual Analog Scale (VAS) and Faces Pain Rating Scale at *p* < 0.05 [F = 6.786 (012*), F = 10.673(002*)], respectively (Table 7).

**Table 7:** Univariate analysis of variance (ANCOVA) and independent samples *t*-test for pain scales in both groups (control and experimental).

Variables		Group		Significance test independent samples *t*-test		Univariate analysis of variance (ANCOVA)
		Control group *n*.31	Experimental group *n*.30			
		M ± SD	M ± SD	T	Sig.	
The visual analog scale (VAS)	Baseline	4.26 ± 2.95	5.70 ± 2.81	3.302	.002*	F = 6.786 *p*-value = .012*
	After Kegel exercise training	3.61 ± 2.50	2.40 ± 2.16			
Faces pain rating scale	Baseline	2.10 ± 1.42	2.20 ± 1.37	2.557	.013*	F = 10.673 *p*-value = .002*
	After Kegel exercise training	1.81 ± 1.35	.83 ± 1.02			

Descriptive statistics, *t*, *t*-test; F, ANCOVA.

* Significant at *p* ≤ 0.05.

Table 8 represents the mean values of all measured variables at baseline and after the Kegel exercise training intervention in both groups. MANOVA showed a significant increase in FSFI in the experimental group after the intervention, with the mean value for the experimental group at baseline being (6.91 ± 1.97) and after (8.00 ± 2.21) at *p* = 0.002. Also, the table illustrates a significant decrease in the Visual Analog Scale and Faces Pain Rating Scale in both groups after the Kegel exercise training intervention at *p* = 0.002 (Table 8).

**Table 8:** Repeated measures MANOVA for all measured variables in both groups (control and experimental).

Domains	Maximum allowed score	Groups				F-value	Intervention-time interaction *p*-value
		Control group *n*.31		Experimental group *n*.30			
		M ± SD	Min-max	M ± SD	Min-max		
Pelvic floor disability index (PFDI-20)							
Baseline	300	67.37 ± 60.14	.00–219.79	76.18 ± 66.70	.00–251.04	3.405	.070
After Kegel exercise training		81.82 ± 59.81	.00–225.00	54.34 ± 74.23	.00–300.00		
The female sexual function index (FSFI)							
Baseline	12	7.14 ± 1.72	2.70–10.20	6.91 ± 1.97	1.80–10.80	10.039	.002*
After Kegel exercise training		6.30 ± 1.74	1.50–9.00	8.00 ± 2.21	2.40–10.80		
The visual analog scale							
Baseline	10	4.26 ± 2.95	1.00–10.00	5.70 ± 2.81	1.00–10.00	10.905	.002*
After Kegel exercise training		3.61 ± 2.50	1.00–10.00	2.40 ± 2.16	1.00–10.00		
Faces pain rating scale							
Baseline	5	2.10 ± 1.42	.00–5.00	2.20 ± 1.37	.00–5.00	6.540	.013*
After Kegel exercise training		1.81 ± 1.35	.00–4.00	.83 ± 1.02	.00–3.00		

Descriptive statistics, *t*, *t*-test, F, MANOVA.

* Significant at *p* ≤ 0.05.

The change between groups corresponded to a high effect size in the Visual Analog Scale, the Female Sexual Function Index, and Faces Pain Rating Scale (Cohen d = 0.84, 0.81, and 0.65, respectively), and a moderate effect size in the Pelvic Floor Disability Index in favor of the experimental group (Table 8).

Table 9 shows Cohen's d effect sizes for the Kegel exercise intervention. The intervention had a medium effect on pelvic floor function (PFDI-20, d = 0.473) and medium-to-large effects on pain reduction (VAS, d = 0.846; Faces Pain Scale, d = 0.655). The strongest impact was on sexual function (FSFI, d = 0.811, large effect). Overall, the intervention meaningfully improved pelvic floor health, sexual function, and pain outcomes.

**Table 9:** Independent samples effect sizes of the Kegel exercise intervention among study groups (control and experimental).

Scales	Cohen's d
Pelvic floor disability index (PFDI-20)	.473
The female sexual function index (FSFI)	.811
The visual analog scale (VAS)	.846
Faces pain rating scale	.655

Cohen's d: a standardized effect size.

A Pearson's Correlation was run to determine the relationship between pre- and post-tests for the four domains (PFDI-20, FSFI, The Visual Analog Scale, and Faces Pain Rating Scale) and participants’ demographic data. There was a positive correlation between PFDI-20 at baseline and women's age, which was statistically significant (r = .254*, *p* = .048). Also, the results revealed that the FSFI was negatively correlated with women's marital status and monthly income at baseline [r = −.264* (040) and r = −.317* (.013) respectively] and positively correlated with women's place of residence after intervention [r = .260* (.043)] (Table 10).

**Table 10:** Correlation between sociodemographic data and pre- and post-tests for the four domains.

Domains		Age *n*.61	Marital status *n*.61	Education *n*.61	Working condition *n*.61	Monthly income *n*.61	Place of residence *n*.61
Pelvic floor disability index (PFDI-20)							
Baseline	r (*p*-value)	.254* (.048)	−.114 (.383)	.028 (.828)	−.159 (.220)	.106 (.416)	−.070 (.589)
After Kegel exercise training	r (*p*-value)	−.118 (.364)	−.130 (.316)	−.014 (.915)	.002 (.989)	.035 (.791)	−.212 (.101)
The female sexual function index (FSFI)							
Baseline	r (*p*-value)	−.174 (.180)	−.264* (.040)	.037 (.774)	.045 (.728)	−.317* (.013)	−.077 (.553)
After Kegel exercise training	r (*p*-value)	.052 (.693)	.186 (.152)	.021 (.874)	−.045 (.732)	−.038 (.770)	.260* (.043)
The visual analog scale							
Baseline	r (*p*-value)	.056 (.667)	−.087 (.506)	−.142 (.275)	.202 (.119)	−.089 (.494)	−.044 (.735)
After Kegel exercise training	r (*p*-value)	−.157 (.227)	−.109 (.401)	−.074 (.568)	.171 (.188)	−.045 (.730)	−.149 (.253)
Faces pain rating scale							
Baseline	r (*p*-value)	.020 (.881)	−.014 (.916)	−.062 (.633)	.122 (.351)	−.031 (.810)	−.098 (.452)
After Kegel exercise training	r (*p*-value)	−.161 (.217)	−.134 (.302)	.046 (.724)	.025 (.846)	−.003 (.981)	.030 (.819)

Pearson's Correlation: r: Correlation coefficient, *p*-value: Sig. (2-tailed).

* Significant at *p* ≤ 0.05.

A Pearson's Correlation was also run to determine the relationship between participants’ previous pregnancy history and pre- and post-tests for the four domains. No statistically significant correlations were found in this study between previous pregnancy history and pre- and post-tests for the four domains (Table 11).

**Table 11:** Correlation between participants’ previous pregnancy history and pre and post-tests for the four domains.

Domains		Miscarriage *n*.61	Type of delivery *n*.61	Number of children *n*.61
Pelvic floor disability index (PFDI-20)				
Baseline	r (*p*-value)	.159 (.221)	.088 (.501)	.001 (.993)
After Kegel exercise training	r (*p*-value)	.072 (.583)	−.151 (.245)	−.096 (.464)
The female sexual function index (FSFI)				
Baseline	r (*p*-value)	−.014 (.917)	−.218 (.091)	−.061 (.642)
After Kegel exercise training	r (*p*-value)	−.009 (.944)	.160 (.218)	−.104 (.423)
The visual analog scale				
Baseline	r (*p*-value)	.072 (.582)	−.164 (.208)	−.080 (.540)
After Kegel exercise training	r (*p*-value)	.108 (.409)	−.194 (.134)	−.158 (.224)
Faces PAIN RATING SCALe				
Baseline	r (*p*-value)	.003 (.981)	−.113 (.386)	−.080 (.540)
After Kegel exercise training	r (*p*-value)	.010 (.939)	−.127 (.329)	−.198 (.127)

Pearson's correlation: r: correlation coefficient, *p*-value: Sig. (2-tailed).

## Discussion

Kegel exercise, also called pelvic floor exercises (PFEs) or pelvic muscle strengthening exercises, is defined as repetitive contractions of the pelvic muscles that control the flow in urination to strengthen these muscles, especially to control or prevent incontinence or to enhance sexual responsiveness during intercourse (22).

The current study's main findings demonstrated that following the Kegel exercise training intervention, the Visual Analog Scale (VAS) and Face Pain Rating Scale significantly decreased in both the control and experimental groups, with the experimental group having significantly lower scores (2.40 ± 2.16,.83 ± 1.02, respectively). The pelvic floor muscles can undergo natural rehabilitation after delivery (38). The rehabilitative ability of the pelvic floor muscles, coupled with the body's natural healing and recovery process over time, may explain the observed difference between the control group's pre-intervention and post-intervention assessment scores. The observed significant difference across the pain scales, which showed significantly lower pain scores in the experimental group, suggests a positive effect of Kegel exercise in decreasing maternal postpartum pain. This finding is in congruence with that of Wang and colleagues, who aimed at using pelvic floor muscle training to relieve persistent lumbopelvic pain after childbirth. The authors recorded success in their rehabilitation program, with participants who received pelvic floor muscle training having a better Numerical Pain Rating Scale score (39). Similar findings were also obtained by El Nahas et al. (40). In contrast, Gutke et al. observed no statistically significant reduction in pain between the treatment and control groups when they studied the efficacy of pelvic floor muscle training exercises for the treatment of chronic postpartum pelvic girdle pain (41). Possible causes for the discrepancy between the results of this study and those of previous studies may include variations in the intervention strategy.

Furthermore, previous research showed that pelvic floor muscle training activities could help manage pelvic floor dysfunction. In a randomized controlled experiment, Johannessen et al. (2020) found that frequent pelvic floor muscle training reduced urine incontinence three months postpartum (42). Woodley et al. also found that pelvic floor muscle training can improve urine and fecal incontinence in prenatal and postnatal women (30). Based on previous literature, it was expected that the experimental group in this study would, to a certain extent, show a significant decrease in pelvic floor disability index scores across its subscales compared to the control group after the intervention period. Contrarily, however, this study's results showed no statistical differences between the experimental and control groups in the total score of pelvic floor dysfunction**.** Potential causes for this discrepancy may include variations in the Kegel exercise technique and/or the assessment instruments used. The length of the intervention is another possible explanation for the discrepancy.

In addition, this study found that the Pelvic Floor Disability Index at baseline was positively correlated with increasing age in women. Age-related decline in pelvic floor muscle function has been linked to an increase in the incidence of pelvic floor disorders.

Based on the results of this study, female sexual function index (FSFI) scores were significantly increased in the experimental group across all domains after the Kegel exercise training intervention. Hadizadeh-Talasaz et al. (1), who conducted a comprehensive review on the impact of pelvic floor muscle training on postpartum sexual function and quality of life, came to the same conclusion. Postpartum women's sexual function was found to be significantly improved with the training of pelvic floor muscles. Exercises targeting the pelvic floor muscles also showed promise in enhancing sexual function. Also, an 8-week pelvic muscle training intervention increased postpartum women's sexual self-efficacy, as described by Golmakani et al. (43).

The levator ani muscle is a broad, thin muscle group situated on either side of the pelvis. It is strengthened through muscular hypertrophy during Kegel exercises, which enhances support (1, 43). Additionally, Kegel exercises improve blood flow to the pelvic and reproductive organs, which boosts lubrication, efferent nerve sensation, and the body's own response to stimulation (44). It improves a woman's sexual experience in terms of orgasmic sensation, duration, and intensity. This justifies the result of the present study, which showed a significant increase in the female sexual function index (FSFI) scores of the experimental group across all domains after the Kegel exercise training intervention.

### Limitations

The present study has several limitations that warrant consideration. It relied on a convenience sample of multiparous women who delivered vaginally, and the relatively small sample size and single-center design may limit the generalizability of the findings to the broader Saudi Arabian population. Cultural factors, including modesty and discomfort in discussing sexual health, could have influenced participants’ responses, despite efforts to foster a supportive and welcoming environment.

Due to cultural sensitivity, participant discomfort, and limited availability of trained assessors, manual muscle testing and vaginal examinations were not feasible; hence, validated functional outcome measures (PFDI-20) were employed as indirect indicators of pelvic floor muscle performance. While pelvic floor rehabilitation may require longer intervention periods, a 6-week intervention was chosen based on feasibility and evidence from prior studies demonstrating short-term benefits in postpartum women. Nevertheless, the lack of long-term follow-up constrains conclusions regarding the sustainability of outcomes, highlighting the need for future research with extended monitoring. Adherence was assessed via self-report, which may introduce bias; subsequent studies are encouraged to implement objective adherence tracking to enhance reliability.

Despite these limitations, the findings provide preliminary but robust evidence supporting the effectiveness of structured health education on Kegel exercises in improving pelvic floor function, alleviating pain, and enhancing sexual function among postpartum women.

## Conclusion

Regarding the impact of Kegel exercise on maternal postpartum pain, pelvic floor dysfunction, and sexual function, the findings of this study showed that female sexual function index (FSFI) scores were significantly increased after the Kegel exercise training intervention. Following the Kegel exercise training intervention, there was a significant decrease in the Visual Analog and Faces Pain Rating Scales. Therefore, the study's null hypothesis is rejected. There were, however, no statistical differences in the total score of pelvic floor dysfunction after the intervention.

### Recommendations

Kegel exercises, which target the pelvic floor muscles and have been shown to strengthen them after pregnancy and childbirth, should be considered a viable treatment option that can be introduced into the clinical and counseling services provided to mothers in Saudi Arabia. Hospital policies should be put in place to establish and maintain pelvic floor exercise training programs that would afford women the opportunity to engage in these exercises before and after pregnancy.

Nurse midwives can also promote a standard of care and practice where changes in the body during pregnancy and after birth are addressed through education, training, treatment, and self-care to decrease problems caused by pelvic floor muscle relaxation following vaginal delivery, improve women's sexual function, and reduce postpartum pain. To aid in the early prevention of pelvic floor disorders in women, nurse midwives should encourage women to improve the frequency and consistency of pelvic floor exercises following delivery. More training for midwives on how to teach the mother the Kegel exercise.

As the intervention of this study was limited to 6 weeks, further research should be carried out to assess the effects of Kegel exercise over a longer period of time and across a more diverse population and parameters.

## Data Availability

The datasets presented in this study can be found in online repositories. The names of the repository/repositories and accession number(s) can be found in the article/Supplementary Material.
