# Supplementary figures and images for: Empowering postpartum women through health education on Kegel exercises: effects on pain, pelvic floor dysfunction, and sexual function
Source: Front Reprod Health. 2026 Feb 2;8:1746383. doi: 10.3389/frph.2026.1746383 (PMC12907388; doi:10.3389/frph.2026.1746383)

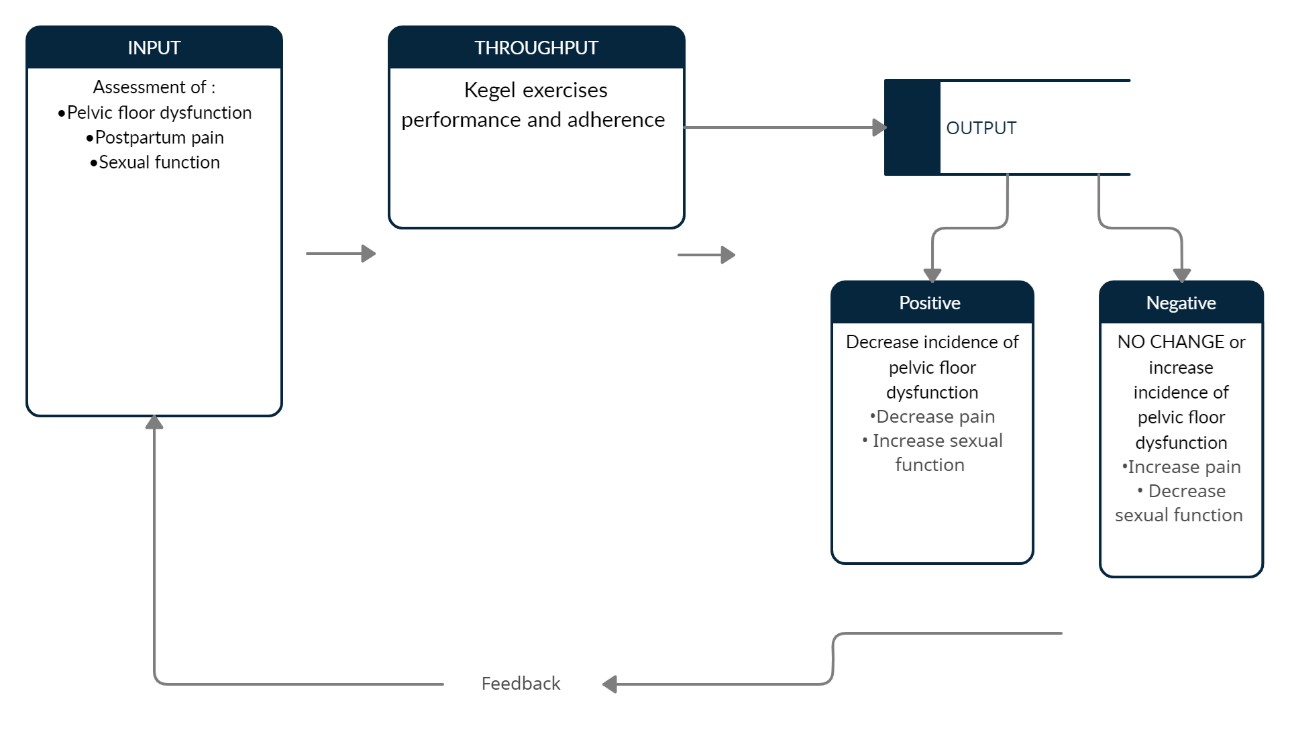

Supplement: Supplementary file 2 [file Image1.jpeg]

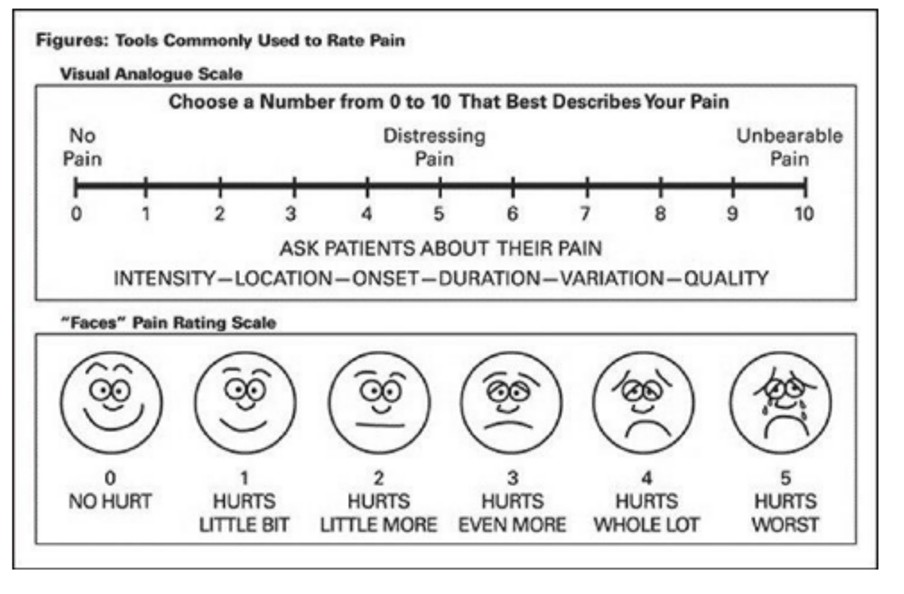

Supplement: Supplementary file 3 [file Image2.jpeg]
