# Supplementary material for: Empowering postpartum women through health education on Kegel exercises: effects on pain, pelvic floor dysfunction, and sexual function
Source: Front Reprod Health. 2026 Feb 2;8:1746383. doi: 10.3389/frph.2026.1746383 (PMC12907388; doi:10.3389/frph.2026.1746383)
Supplement: Supplementary file 4 [file Table1.docx]

**Cover Letter**

Dear Respected Editors,

I would like to express my sincere appreciation for your continuous efforts in advancing scientific knowledge. On behalf of my co-authors, I am pleased to submit our original research article entitled “**Empowering Postpartum Women through Health Education on Kegel Exercises: Effects on Pain, Pelvic Floor Dysfunction, and Sexual Function**” for your kind consideration.

We believe that this study makes a meaningful contribution to existing literature by addressing a significant maternal health issue. The research evaluates the clinical effectiveness of Kegel exercises in managing postpartum pain, pelvic floor dysfunction, and sexual function, providing evidence-based insights that can inform maternal health care practice and promote women’s quality of life.

After careful review of relevant journals, we selected *your journal* because its scope aligns closely with the objectives and implications of our study. We are confident that this paper will be of interest to your readership and will contribute to the advancement of evidence-based maternal health practice.

Thank you very much for your time and consideration. We look forward to the opportunity to contribute to your prestigious journal.

**Sincerely,***Dr. Neama Hantira* **(On behalf of all co-authors)**
