# Supplementary material for: Empowering postpartum women through health education on Kegel exercises: effects on pain, pelvic floor dysfunction, and sexual function
Source: Front Reprod Health. 2026 Feb 2;8:1746383. doi: 10.3389/frph.2026.1746383 (PMC12907388; doi:10.3389/frph.2026.1746383)
Supplement: Supplementary file 1 [file Datasheet1.pdf]

Kingdom of Saudi Arabia

المملكة العربية السعودية

Ministry of National Guard - Health Affairs

وزارة الحرس الوطني - الشؤون الصحية

King Abdullah International Medical Research Center (KAIMRC)

(84) 94456 1515 irb@ngha.med.sa

National Committee of Bioethics Registration No: H-01-R-005

IRB Office

E-CTS Ref. No. JED-22-427780-77010

IRB Approval No.: **IRB/1251/22**
Study Number: **SP22J/060/05**
Study Title: **Impact of Kegel exercise on Maternal Postpartum Pain, Pelvic Floor Dysfunction and Sexual Function among Women in Jeddah, Saudi Arabia**
Study Sponsor: **Non Grant**
IRB Approval Date: **30 June 2022**
IRB Review Type: ☐ **Expedited Review** ☑ **Full Board**
Study site(s): **Central Region**

KAIMRC APPROVED IRB

Dear **Dr. Nasreen Abdulmannan**
Section Head, Plastic Surgery, KAMC
Ministry of National Guard – Health Affairs

Sub-investigator/s: Jawaher Hazza Alharbi, Dr. Neama Hantira

After reviewing your submitted research proposal/protocol and related documents, the **IRB has APPROVED** the submission. The approval includes the following related documents:

Document/Title	Version	Date
Research Proposal	01	30 June 2022
Data Collection	01	30 June 2022
Informed Consent Form	01	30 June 2022

The approval of the research study is valid for **one year** from the above approval to expiration date.

Terms of Approval:

- **Annual Reports:** An Annual report must be submitted for approval to avoid termination/suspension of your research.
- **Financial report:** If your study is funded project, details financial report should be submitted with the scientific report.
- **Final Report:** After completion of the study, a final report must be forwarded to the IRB.
- **Retention of original data:** The PI is responsible for the storage and retention of original data pertaining to the project for a minimum of five years.
- **Reporting of adverse events or unanticipated problems:** The PI is responsible to report any serious or unexpected adverse events or unanticipated problems, which could involve any risk to participants or others, or any event on incidents that may have impact on the research or participants.
- **Biological samples:** No biological samples to be shipped out of the Kingdom of Saudi Arabia without prior IRB approval.
- **Participant incentives:** No financial compensation or gifts to be given to participants without prior IRB approval.
- **Storage of biological samples:** All biological samples collected for the purpose of this research must be stored in the KAIMRC related repository.
- This IRB approved research study must not contradict with any Saudi law including but not limited to the Saudi Law of Ethics of Research on Living Creatures and its Implementing Regulations.
- You will need to resubmit the proposal to the IRB for review and re-approval before implementing any changes to the approved proposal.
- It is possible that the IRB may decide that the proposed new changes may exclude the proposal from being accepted for exempt review.
- It is your responsibility to safely store the data collected.
- Please note that phone based surveys are not permitted.
- If your approved proposal requires access to Bestcare, please write to the IRB informing them of the name of the designated data collector and exactly define the period requested for collecting data. Do not start the data collection until an approval memo is issued from the IRB giving permission to that collector to start accessing Bestcare for the duration of the project and after signing a confidentiality agreement.
- To submit a copy of your manuscript to the IRB.

Prof. Hamdan Al Jahdali
A/Chairman, Institutional Review Board (IRB)
Ministry of National Guard - Health Affairs
HJ/FAG

18 JUL 2022
